# Appropriate glycated haemoglobin testing frequency to monitor patients with type 2 diabetes

**DOI:** 10.4102/phcfm.v18i1.5614

**Published:** 2026-09-29

**Authors:** Wade Thompson, Tom Perry, Guillaume Grenet, Ken Bassett, Anshula Ambasta

**Affiliations:** 1 Department of Anaesthesiology, Pharmacology, and Therapeutics, Faculty of Medicine, University of British Columbia, Vancouver, British Columbia, Canada

**Keywords:** blood glucose, diabetes mellitus, glycated haemoglobin, physiologic monitoring, HBA1c testing frequency, older people

## Abstract

Glycated haemoglobin (HbA1c) is used to monitor glycaemic control among patients with type 2 diabetes (T2DM). Glycated haemoglobin represents a 3-month glycaemic average, with the most recent 6 weeks of glycaemia having the greatest influence. Analytical and biological variation introduce imprecision into any single HbA1c, where a result of 7.5% may represent a ‘true’ value between 6.8% and 8.2%. This Therapeutics Letter evaluates the most appropriate interval for monitoring HbA1c in T2DM. Evidence was synthesised from a pragmatic randomised controlled trial (RCT) and three large observational studies. In the RCT, 94% of patients monitored every 6 months maintained good glycaemic control compared to 92% of those monitored every 3 months. Observational data indicated that 3-month testing resulted in a clinically unimportant difference in HbA1c compared with 6-month testing. Furthermore, patients tested 2–3 times annually were as likely to achieve personalised goals as those tested four times per year. Testing more than four times annually was associated with a lower probability of achieving targets. For patients with stable T2DM meeting glycaemic goals, monitoring HbA1c every 6 months is as effective as every 3 months and could be contemplated for many patients following a discussion of patient goals and preferences. Frequent testing may be reserved for periods of medication adjustment, changes in health status or when targets are unmet. Reducing testing frequency in stable patients minimises low-value data, reduces the carbon footprint of lab testing and improves patient convenience without compromising clinical outcomes.

**Vignette:** *Your 68-year-old patient with type 2 diabetes has taken metformin 1000 mg twice daily, and empagliflozin 10 mg once daily for the last 6 years. Over this interval, you checked his HbA1c every 3 months – 24 times, so far. It has ranged from 6.7% to 7.4% for the last two years*. **Should you continue to re-test the HbA1c every 3 months?**

## Summary and conclusions

- The optimal glycated haemoglobin (HbA1c) monitoring interval to improve clinical outcomes for people with type 2 diabetes (T2DM) has not been established from controlled clinical studies.
- Observational studies suggest that monitoring HbA1c every 3 months is not better than less frequent monitoring (e.g. every 6 months) to achieve glycaemic targets.
- Be cautious about over-interpreting any single HbA1c result or a small change in HbA1c, because of imprecision around any HbA1c result.
- As with other tests, ordering an HbA1c makes sense only if a result is likely to lead to a change in care.

## Glycated haemoglobin biochemistry and physiology

Glucose attaches to haemoglobin inside red blood cells (RBCs), creating glycated (sugar-coated) haemoglobin.^1^ The proportion of haemoglobin that is glycated is closely correlated with the average concentration of glucose in the bloodstream over time and is reported as HbA1c.^1,2^ Glycated haemoglobin does not require fasting or a timed sample.

Because RBCs live about 120 days (range 70–140 days)^3,4^ an HbA1c test provides an estimate of a person’s average blood glucose level over the previous 3 months.^2^ However, blood glucose levels in the 6 weeks immediately before an HbA1c test have a greater influence on the result than levels in the first half of a 3-month interval.^5^

Any condition that increases the lifespan of an RBC or reduces RBC turnover can result in misleadingly elevated HbA1c results – for example, untreated iron deficiency and other nutritional deficiency anaemias, which slow erythropoiesis and compensate by increasing the RBC lifespan.^5^ Conversely, conditions that shorten RBC lifespan, such as acute or chronic bleeding and advanced kidney disease, may result in misleadingly low HbA1c results.^5^

More important for patient care is typical biological (physiologic) and analytical (laboratory) variability that affects the interpretation of patient monitoring.^6^ For any single measurement of HbA1c, the imprecision (from both biological and analytic variability) of a result may be such that an HbA1c of 7.5% could reflect a ‘true HbA1c’ somewhere between 6.8% and 8.2%.^6^ *For this reason, it is a mistake to weigh heavily any single HbA1c value or a small change in HbA1c over time. Rather, it is important to consider the trend of HbA1c values along with the imprecision that exists around a single value*.

## How often should we check glycated haemoglobin to monitor people with type 2 diabetes?

Because it takes time for HbA1c to reflect any change in glucose levels, it does not usually make sense to check HbA1c within 3 months of a previous test. Several studies have examined the impact of different HbA1c monitoring intervals on achieving HbA1c goals in people with T2DM, although not on clinical outcomes. The only randomised controlled trial (RCT) on this topic was a pragmatic trial conducted in people with T2DM and an HbA1c < 7.5% (*N* = 2215) and followed for 18 months.^7^ This evaluated glycaemic control from testing HbA1c every 3 months versus 6 months. In the ‘every 3 months’ group, 92% remained in good control, versus 94% in the ‘every 6 months’ group. The investigators concluded that there was no clinically relevant difference between every 3 months versus every 6 months HbA1c testing.

In two non-randomised observational studies (*N* = 79 409 and *N* = 19 908), testing HbA1c every 3 months versus every 6 months was associated with a very small decrease in HbA1c: about 3% relative reduction over 2–4 years.^8,9^ This would correspond to a change in mean HbA1c from 7% to about 6.8% – an accomplishment of no clinical relevance.

Finally, a large cohort study (*N* = 20 690) followed people with T2DM for up to 4 years. Even in those whose HbA1c was considered ‘sub-optimal’, testing every 6 months achieved HbA1c goals as well as testing every 3 months.^10^ Undergoing only a single annual test or more than four tests per year was both associated with a lower probability of achieving a target HbA1c.

*People having 2–3 HbA1c tests annually were as likely to achieve a personalised goal as those who underwent four tests per year*. Thus, available evidence casts doubt on any clinical advantage of testing every 3 months versus less frequently.

## No study has assessed how the frequency of glycated haemoglobin testing influences clinical outcomes that are important to people with type 2 diabetes

In South Africa, guidelines for the management of T2DM, which accounts for more than 90% of all diabetes cases in South Africa, are provided by the Society for Endocrinology, Metabolism and Diabetes of South Africa (SEMDSA).^11^ Recommendations from SEMDSA guidelines for the glycaemic control target and HbA1c monitoring frequency (6 monthly in well controlled patients) are in line with those from the Canadian guidelines,^2^ the British Columbia guidelines,^12^ the United Kingdom (UK) National Institute for Health and Care Excellence (NICE)^13^ and the American Diabetes Association (ADA),^14^ which make the following suggestions for monitoring of T2DM:

- Check HbA1c every 3 months when blood glucose targets are not met, or when changes in diabetes management are underway.
- Check HbA1c every 6 months when blood glucose goals are met, and management is stable.

Research studies supporting longer HbA1c testing intervals in well-controlled T2DM cite that too frequent testing may lead to unnecessary regimen changes, which may further contribute to adverse effects, including hypoglycaemia, a greater treatment burden and higher healthcare costs.^15,16^ The available evidence reviewed above provides additional nuance to these recommendations and supports the notion that less frequent testing (e.g. every 6 months) could be considered for many patients after a discussion of patient preferences and goals. Reducing unnecessary or low-value tests is suggested to have the highest potential to reduce the carbon footprint of pathology testing.^17^ For patients, this means less travel and inconvenience, as well as fewer venepunctures. For clinicians, it may also mean fewer data to handle in a busy day.^18^

*These considerations do not apply to patients with unstable trajectories caused by changes in health status or concomitant treatments, or situations where treatment is being adjusted to respond to severe hyperglycaemia. In these situations, more frequent HbA1c testing may be appropriate*.

## What is the purpose of glycated haemoglobin monitoring?

The utility of HbA1c monitoring is to achieve glycaemia in a range that meets clinician and patient goals for diabetes care. Glycaemic goals should be individualised. For most people with T2DM, it is reasonable to target moderate HbA1c goals from 7% to 8%,^19^ considered as the average of several measurements – rather than any single test. More relaxed goals may be appropriate in frail, older adults, whereas more stringent goals (e.g. HbA1c < 7%) might be reasonable in people with recent onset T2DM, low risk of adverse effects from treatment, low treatment burden and longer life expectancy.^19,20,21,22^

In T2DM, the importance of achieving HbA1c targets < 7% has been challenged^23^ despite guideline recommendations.^22,24^ This is because targeting HbA1c at < 7% versus < 8% does not reduce the incidence of premature death or cardiovascular events, but it does increase hypoglycaemia risk.^19^ Recent large RCTs of the sodium glucose cotransporter-2 inhibitors and glucagon-like peptide-1 receptor agonists (GLP-1RA) achieved improvement in outcomes meaningful to patients – such as major adverse cardiovascular events – with mean achieved HbA1c in the mid 7% range.^25,26,27^

Achieving an HbA1c < 7% has been shown to reduce microvascular outcomes, although these improvements are primarily in surrogate markers such as albuminuria rather than clinically important outcomes such as vision loss or dialysis.^19,23^ Achieving a strict low target HbA1c can require complex and burdensome treatment regimens. Thus, individualised care should balance possible benefits with the burden of medications and adverse effects.

**Vignette resolution:** *Your patient’s pharmacological regimen is stable, and his HbA1c reflects glycaemic control within a range clinically appropriate at age 68. When you suggest reducing the frequency of HbA1c tests to every 6 months, he is relieved to undergo fewer blood draws and reduce his trips to the laboratory. This inspires you to consider other patients who could benefit similarly and reassess the frequency with which you requisition HbA1c and other common laboratory tests*.
